# Supplementary material for: Antimicrobial Profile of Actinomycin D Analogs Secreted by Egyptian Desert Streptomyces sp. DH7
Source: Antibiotics (Basel). 2021 Oct 18;10(10):1264. doi: 10.3390/antibiotics10101264 (PMC8532959; doi:10.3390/antibiotics10101264)
Supplement: Supplementary file 1 [file antibiotics-10-01264-s001.zip › antibiotics-1351043-SI.pdf]

antibiotics

MDPI

Article

Antimicrobial Profile of Actinomycin D Analogs Secreted by Egyptian Desert *Streptomyces* sp. DH7

Dina H. Amin [1,*], Hayam A. E. Sayed [1], Ahmed M. Elissawy [2,3], Dina E. EL-Ghwas [4] and Abdel Nasser B. Singab [2,3]

1 Department of Microbiology, Faculty of Science, Ain Shams University, Cairo 11566, Egypt; hayam84@sci.asu.edu.eg
2 Department of Pharmacognosy, Faculty of Pharmacy, Ain Shams University, Cairo 11566, Egypt; aelissawy@pharma.asu.edu.eg (A.M.E.); dean@pharma.asu.edu.eg (A.N.B.S.)
3 Center of Drug Discovery Research and Development, Faculty of Pharmacy, Ain Shams University, Cairo,11566, Egypt
4 Pharmaceutical Industries Researches Division, Department of Chemistry of Natural and Microbial Products, National Research Centre, Giza 12622, Egypt; dinaelghwas7781@yahoo.com
* Correspondence: dina.hatem@sci.asu.edu.eg

Supplementary Materials

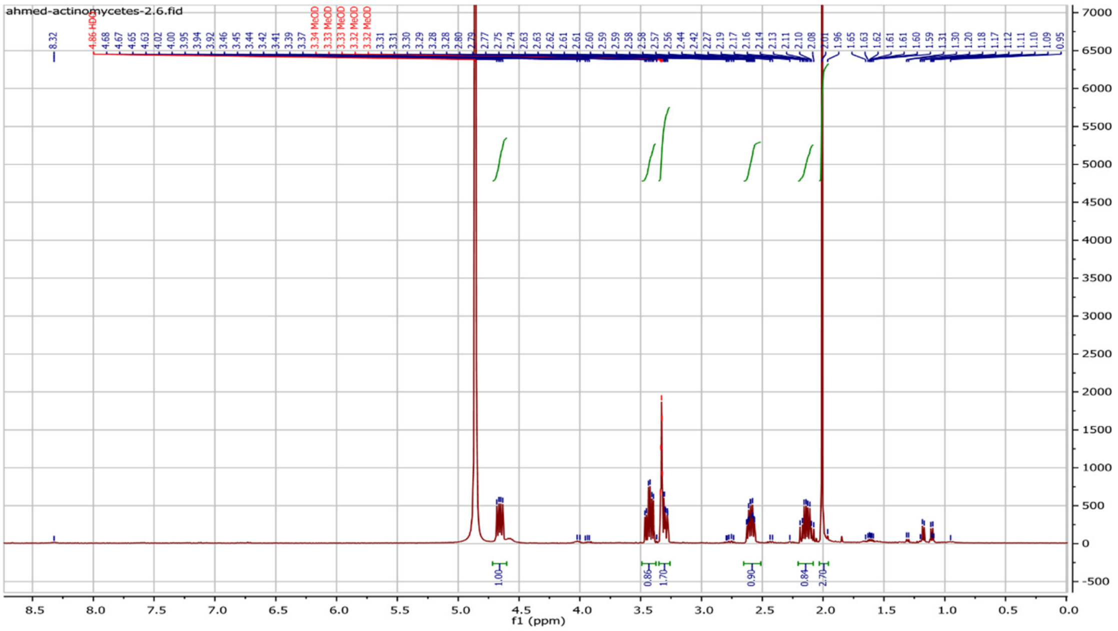

Figure S1. ^{1}H NMR spectrum of compound 1.

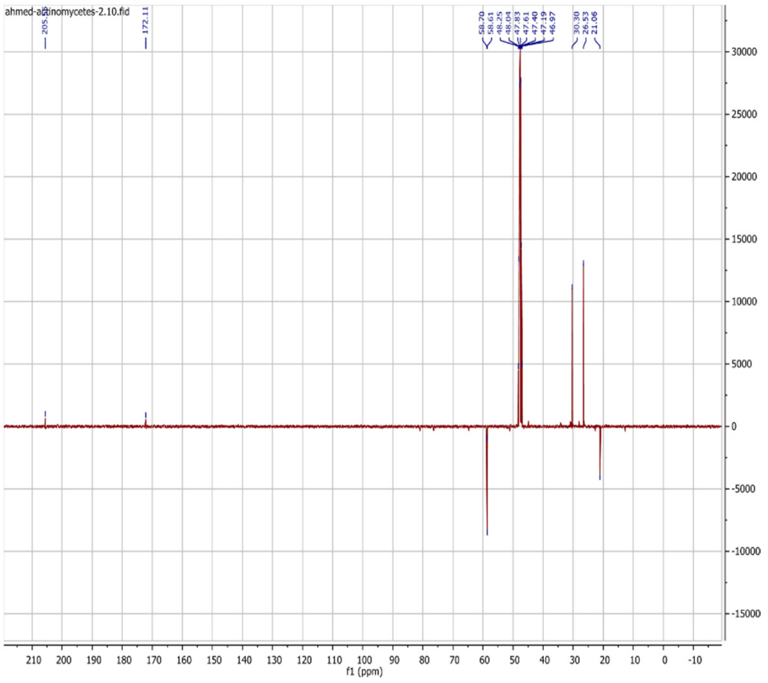

Figure S2. APT NMR spectrum of compound 1.

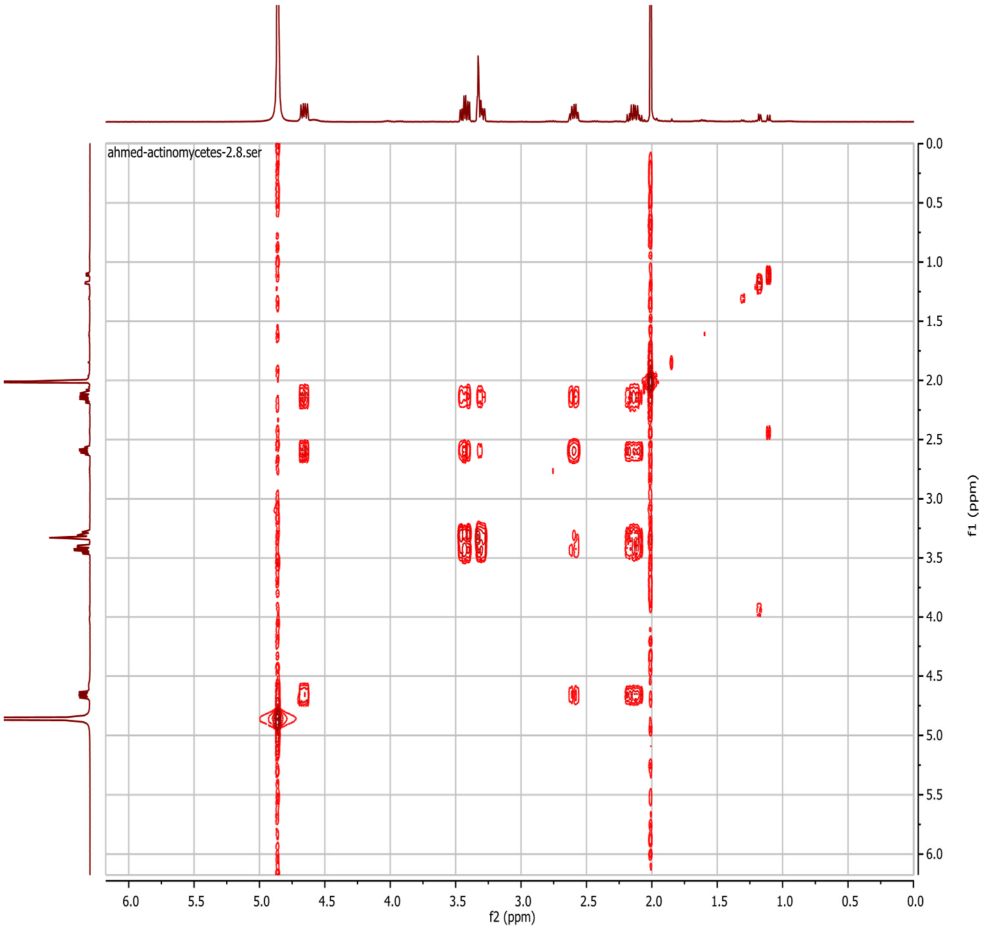

Figure S3. COSY NMR spectrum of compound 1.

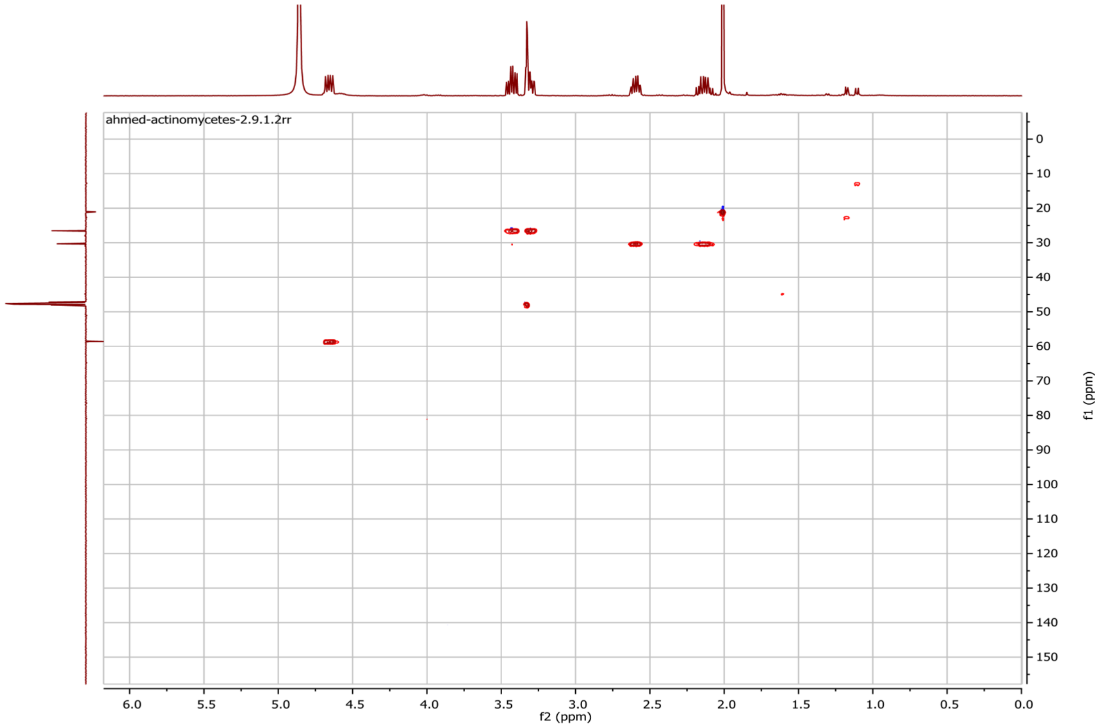

Figure S4. HSQC NMR spectrum of compound 1.

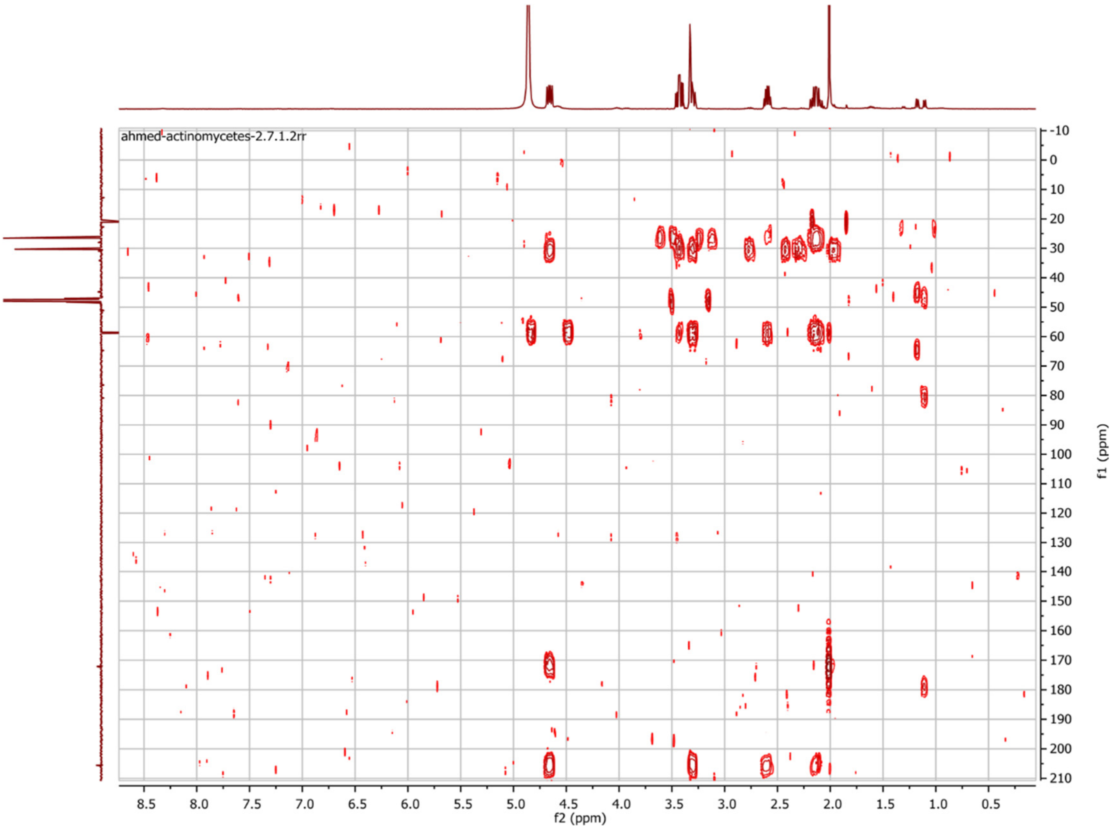

Figure S5. HMBC NMR spectrum of compound 1.

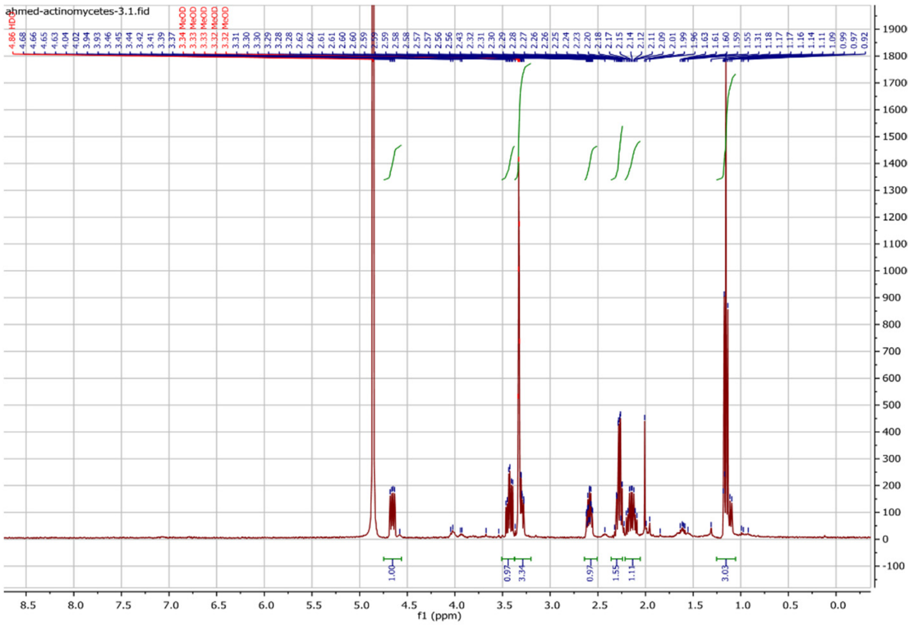

Figure S6. 1H NMR spectrum of compound 2.

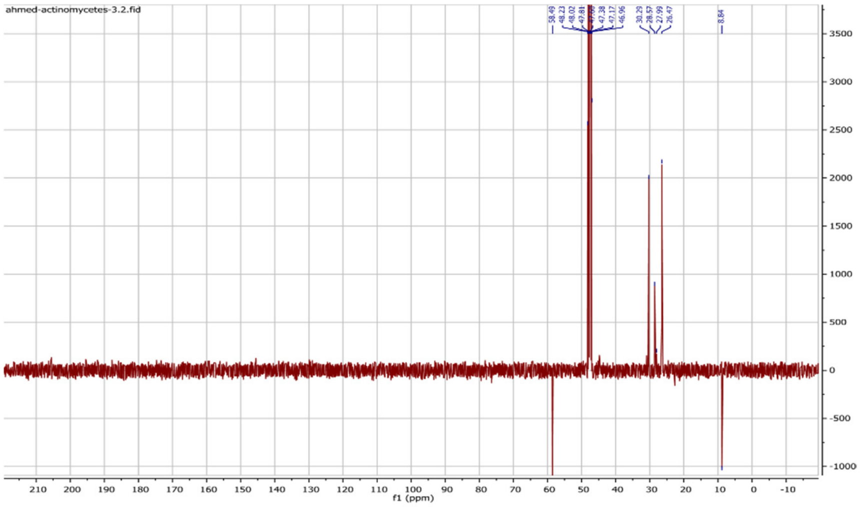

Figure S7. APT NMR spectrum of compound 2.

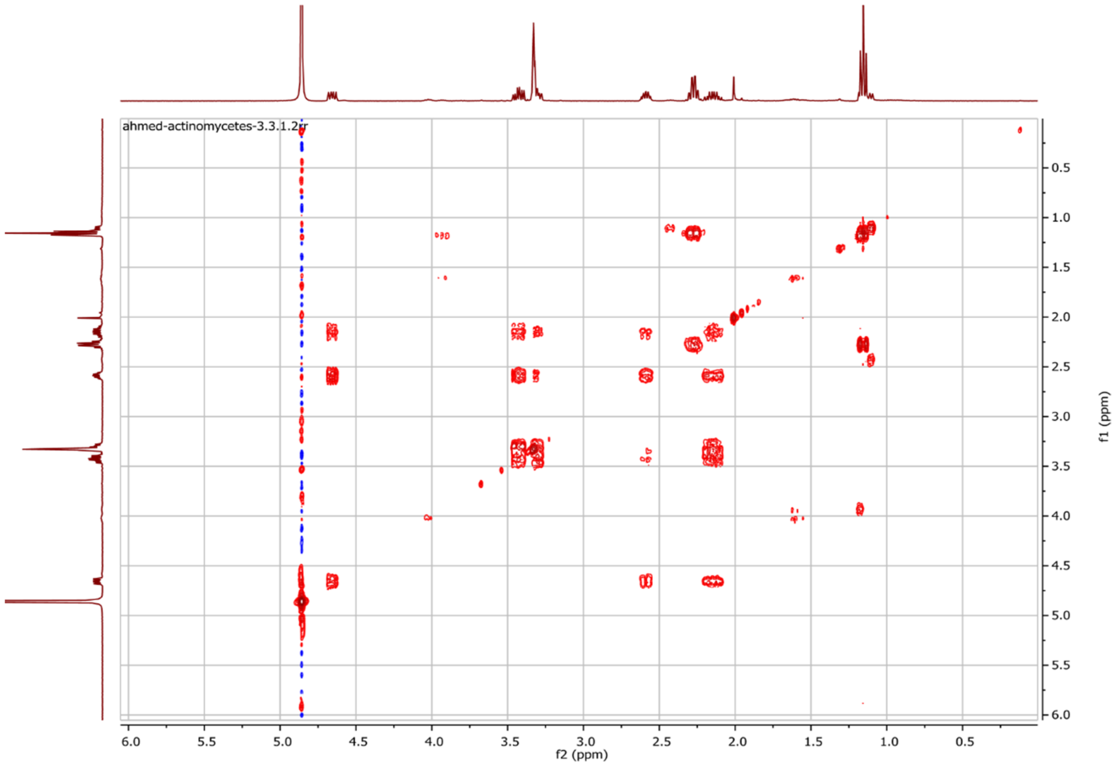

Figure S8. COSY NMR spectrum of compound 2.

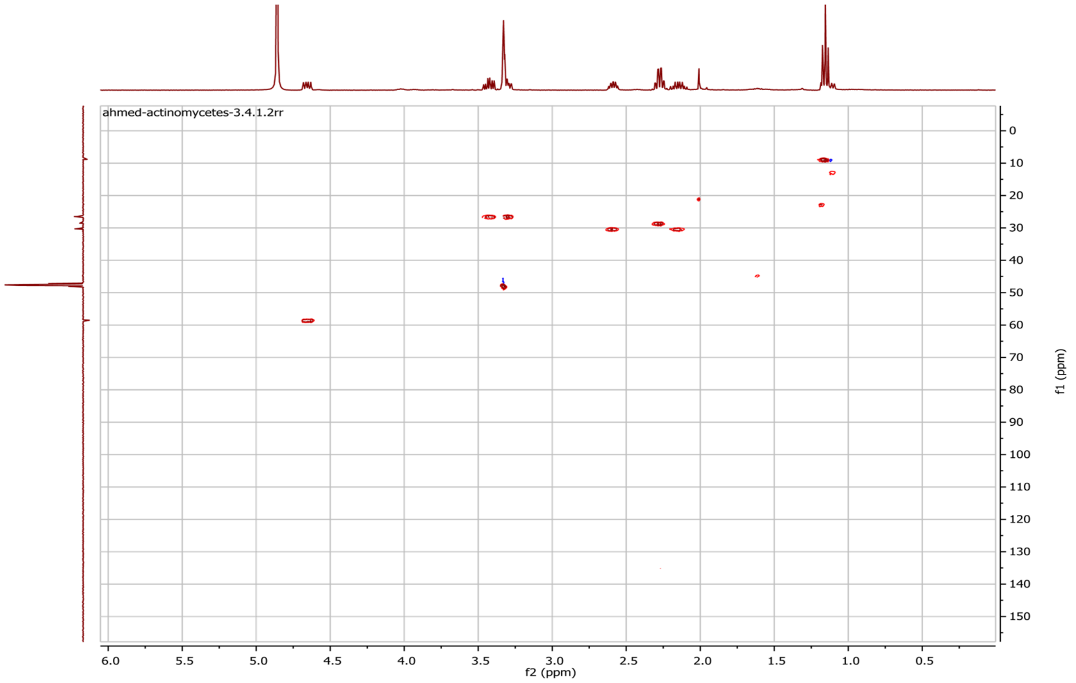

Figure S9. HSQC NMR spectrum of compound 2.

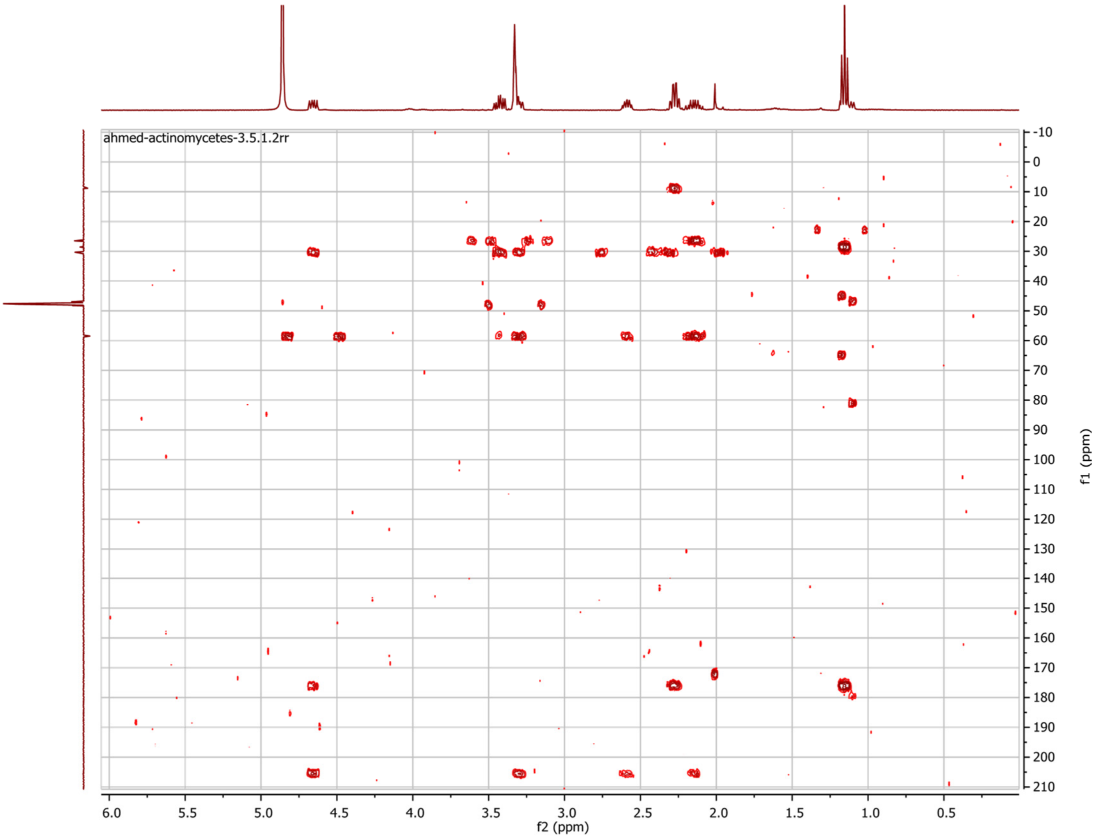

Figure S10. HMBC NMR spectrum of compound 2.

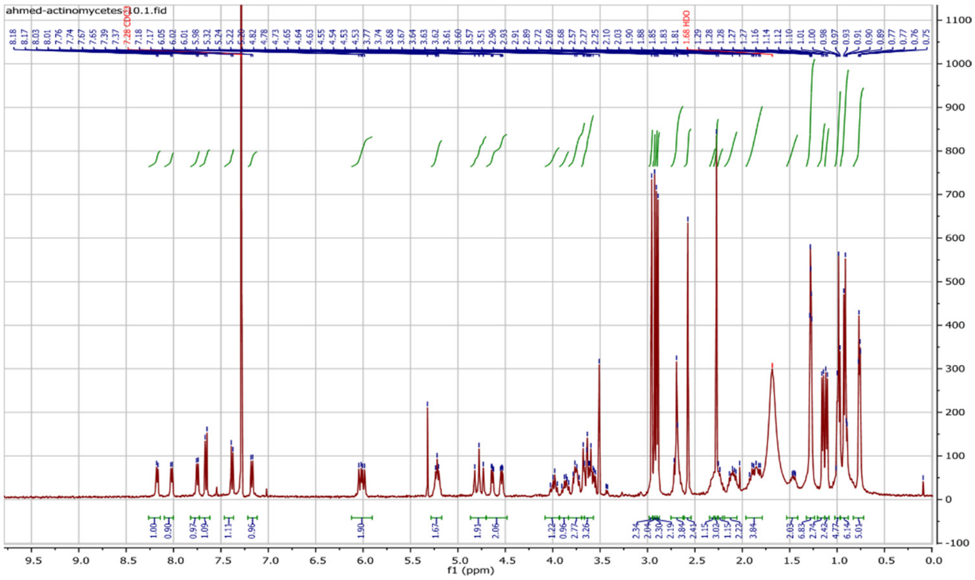

Figure S11. 1H NMR spectrum of compound 3.

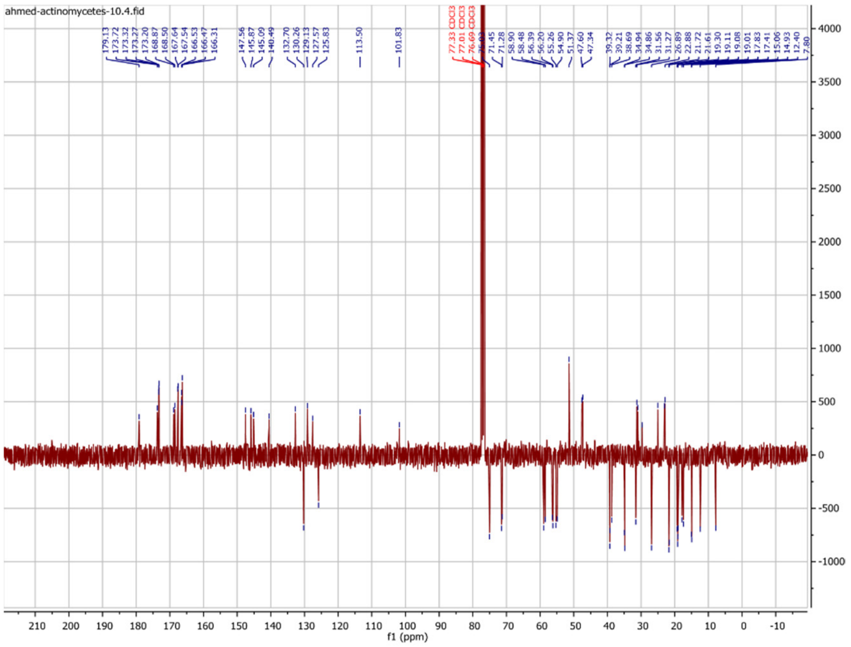

Figure S12. APT NMR spectrum of compound 3.

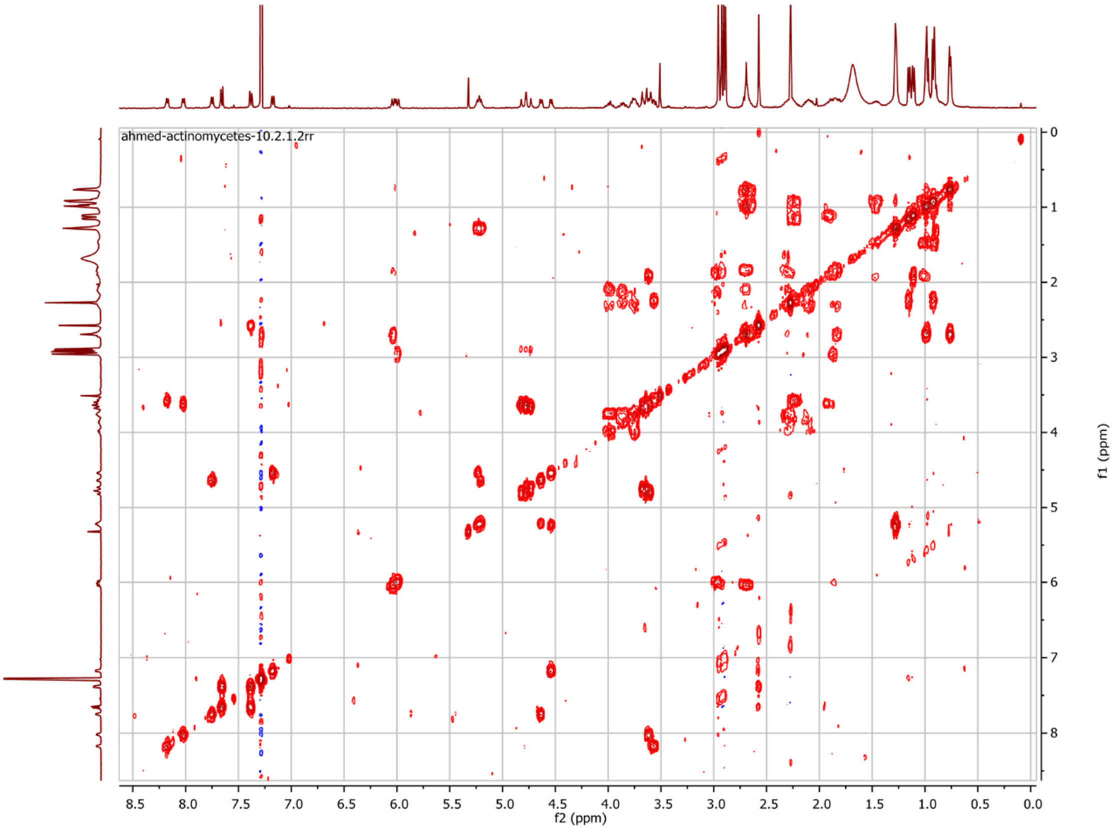

Figure S13. COSY NMR spectrum of compound 3**.**

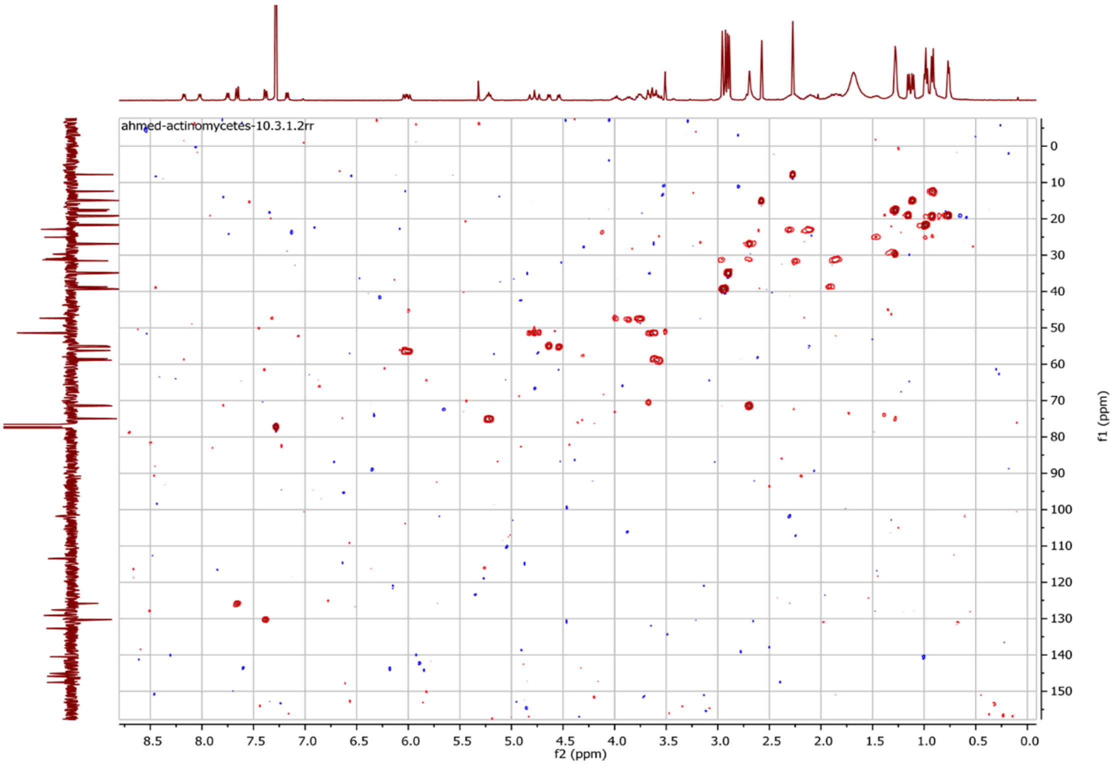

Figure S14. HSQC NMR spectrum of compound 3.

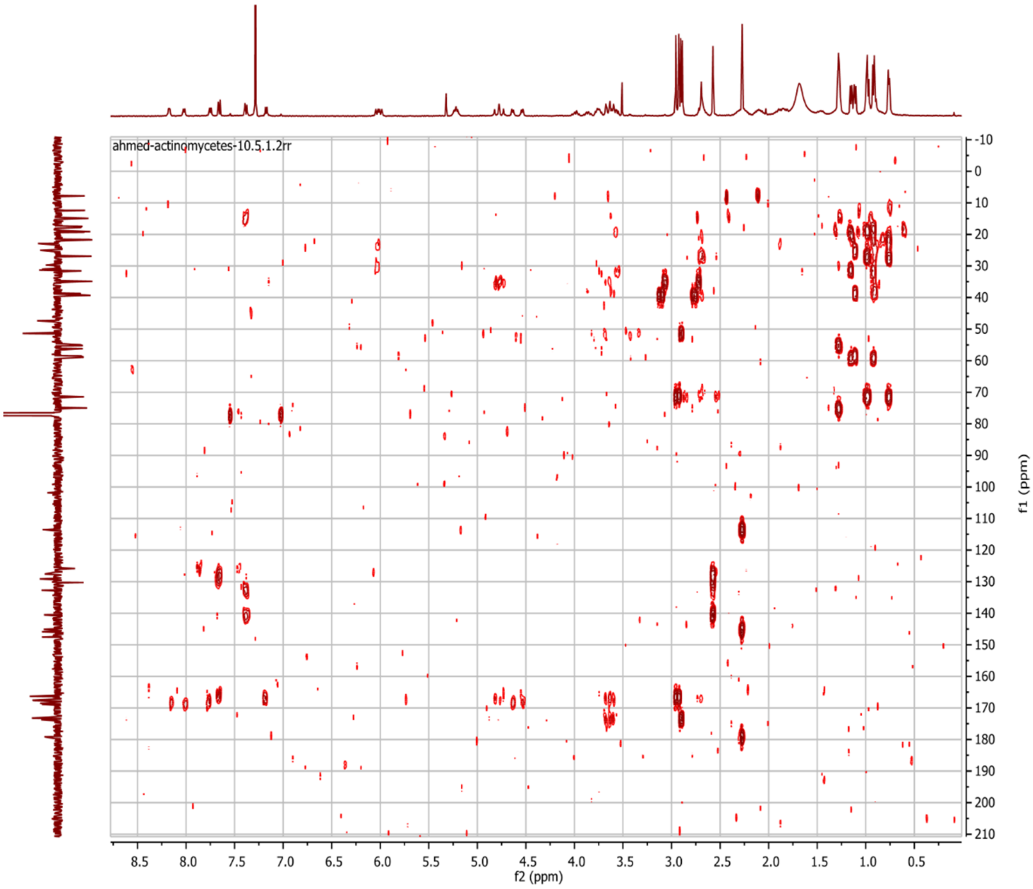

Figure S15. HMBC NMR spectrum of compound 3.

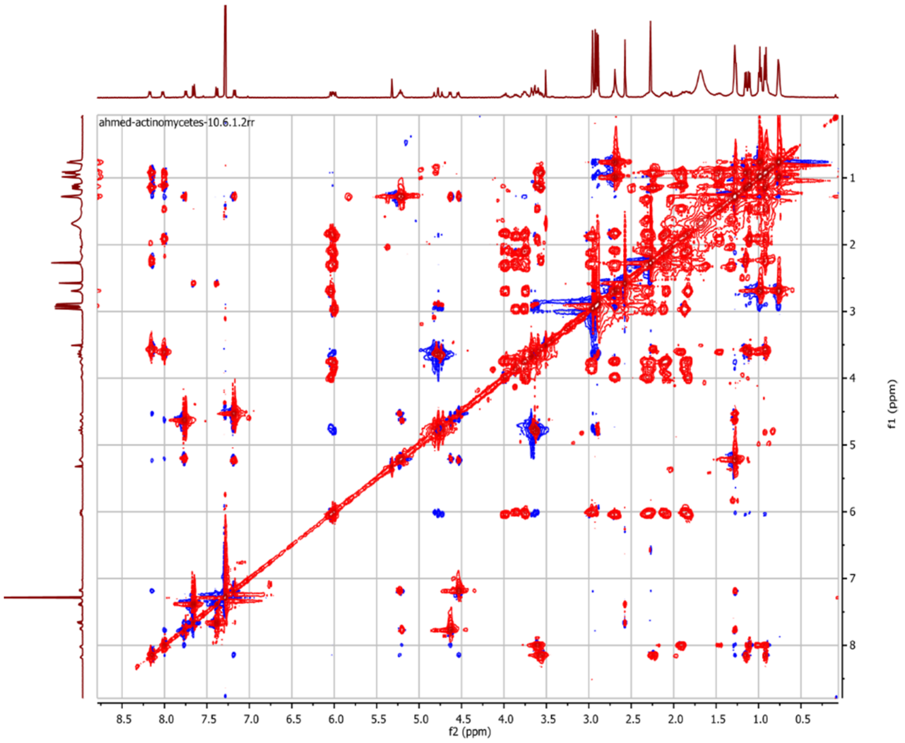

Figure S16. TOCSY NMR spectrum of compound 3.

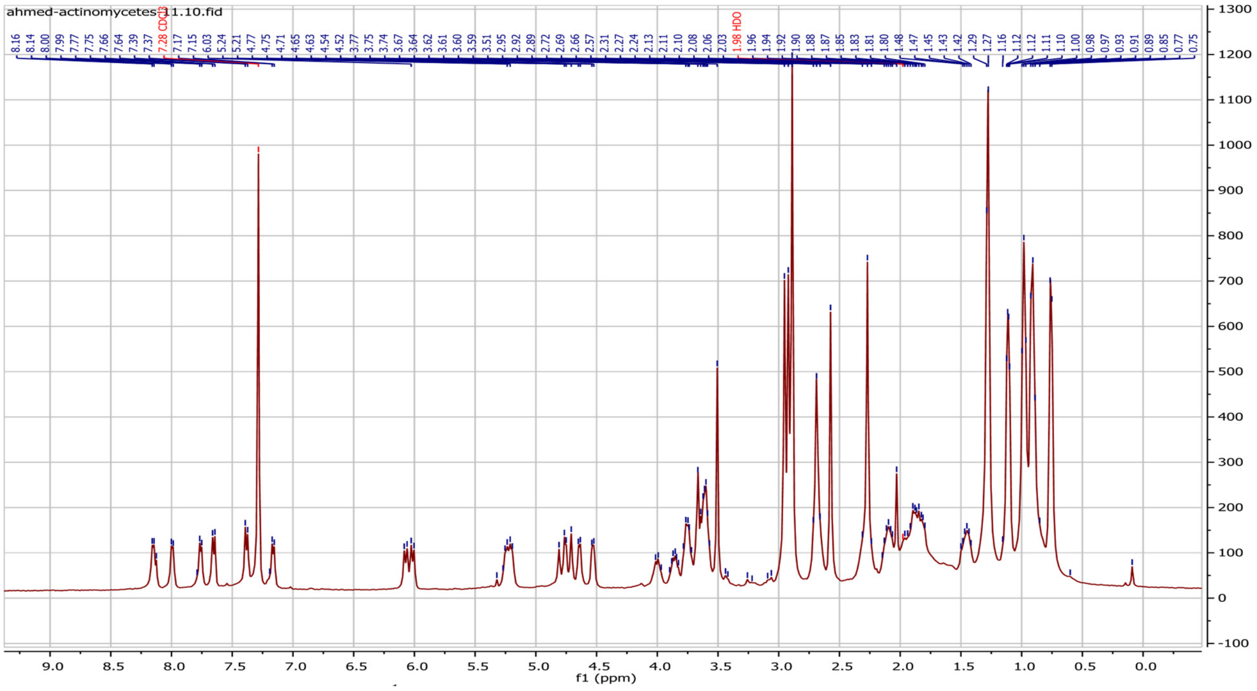

Figure S17. 1H NMR spectrum of compound 4.

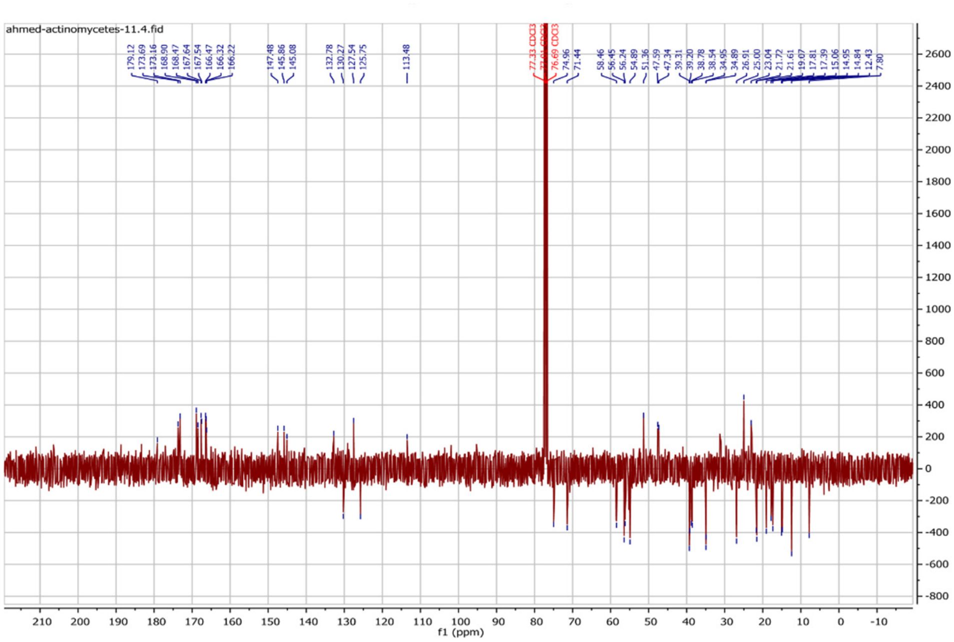

Figure S18. APT NMR spectrum of compound 4.

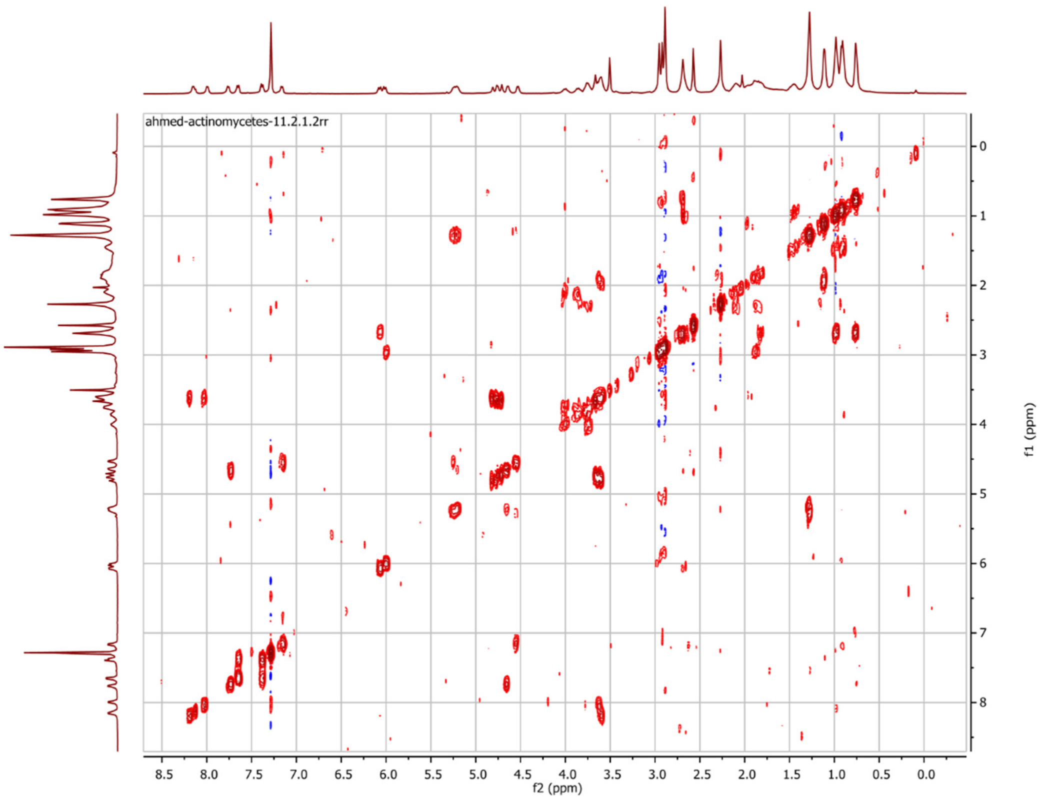

Figure S19. COSY NMR spectrum of compound 4.

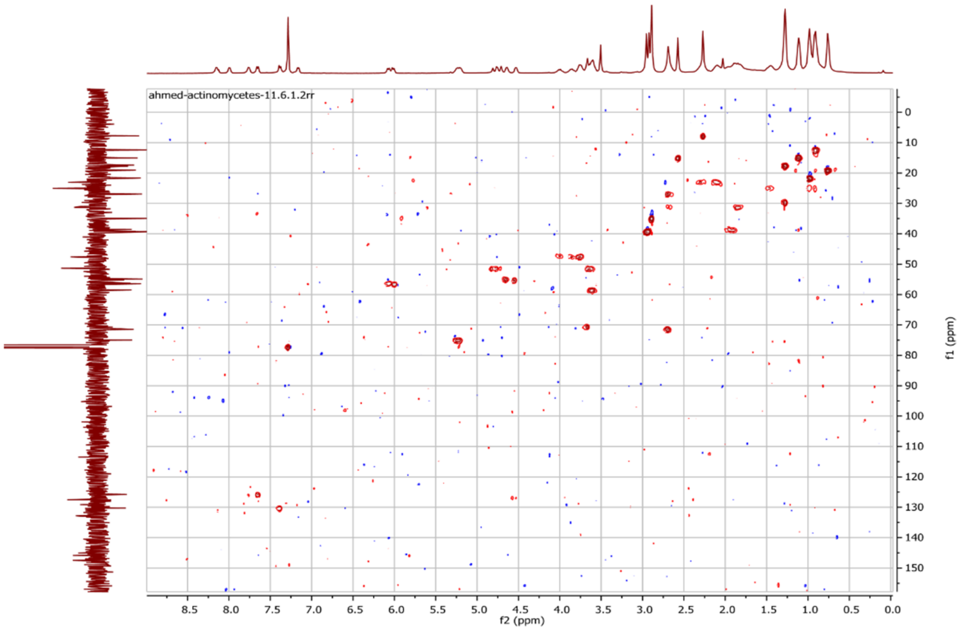

Figure S20. HSQC NMR spectrum of compound 4.

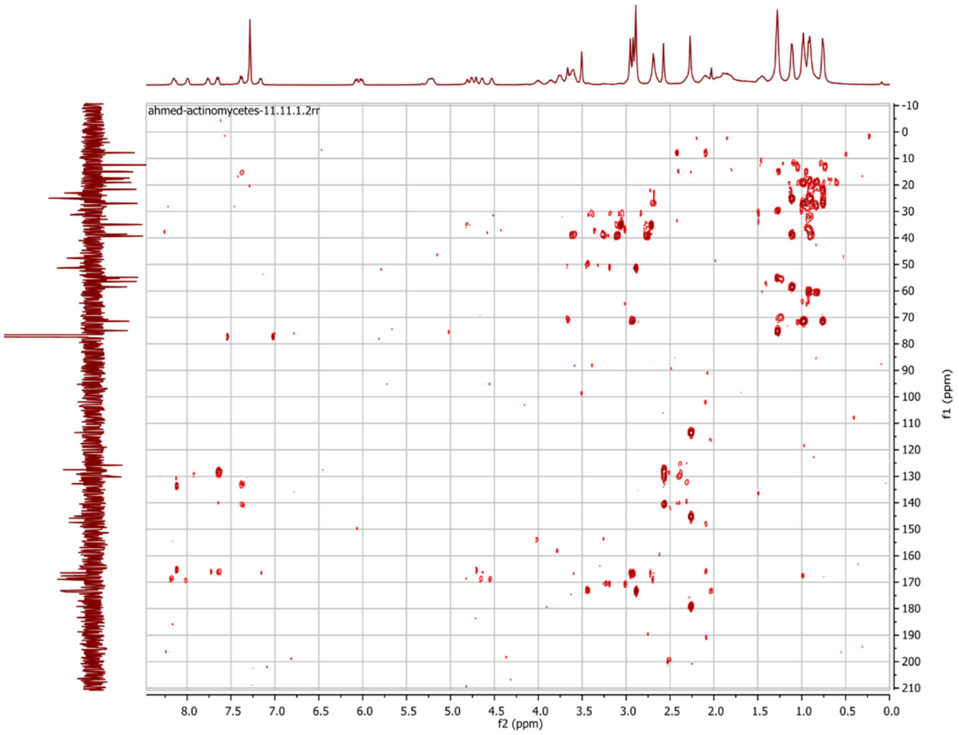

Figure S21. HMBC NMR spectrum of compound 4.

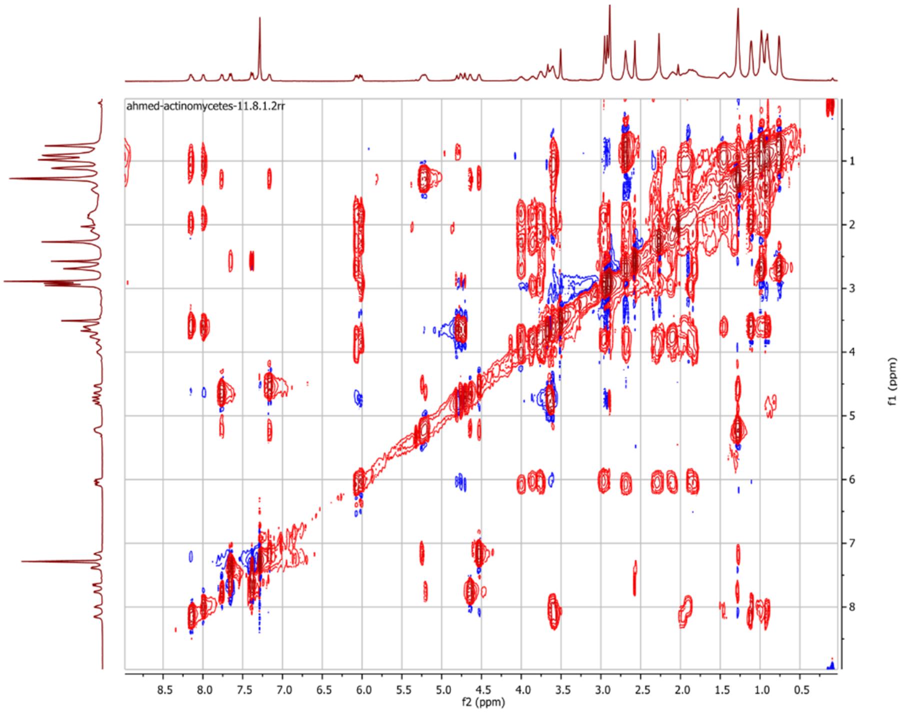

Figure S22. TOCSY NMR spectrum of compound 4.

Table S1. Identification of bacterial strains using MALDI/TOF (Matrix-assisted laser desorption ionization/ time-of-flight), showing score values.

Isolate's code	Organism Identification	Score value	Category	Symbol	NCBI Identifier
30	*Staphylococcus aureus*	2.12	A	++	1280
34	*Staphylococcus aureus*	2.18	A	++	1280
35	*Staphylococcus aureus*	2.17	A	++	1280
37	*Staphylococcus aureus*	2.27	A	++	1280
39	*Staphylococcus aureus*	2.13	A	++	1280
43	*Staphylococcus aureus*	2.37	A	+++	1280
55	*Staphylococcus aureus*	2.32	A	+++	1280
60	*Staphylococcus aureus*	2.17	A	++	1280

Table S2. Antimicrobial susceptibility profile of *Staphylococcus aureus* strains used in this study.

**Isolates** **Antibiotics**		**30**	**34**	**35**	**37**	**39**	**43**	**55**	**60**
Penicillins	Penicillin(P,10units)	R	R	R	R	R	R	R	R
	Oxacillin(OX,30)	R	R	R	R	R	I	R	R
	Cefoxitin(FOX, 30µg)	R	R	R	R	R	R	R	R
Glycopeptides	Vancomycin(V,30 µg)	R	R	S	S	S	R	R	R
Aminoglycosides	Gentamicin (CN,10µ)	R	R	R	R	R	R	R	R
	Amikacin(AK,30µg)	R	S	R	R	S	R	R	R
	Tobramycin(TOB,10µg)	R	R	R	R	R	R	R	R
Macrolides	Azithromycin (AZM,15µg)	R	R	S	R	S	R	R	R
	Clarithromycin(CLR,15 µg	R	R	R	R	S	R	R	R
	Erythromycin(E,15 µg)	R	R	R	R	S	R	R	R
Tetracyclines	Tetracycline(TE,30 µg)	R	S	R	R	R	R	R	R
Fluoroquinolones	Ciprofloxacin(CIP,5 µg)	R	R	R	R	R	R	R	R
	Levofloxacin(LEV,5 µg)	R	R	R	R	R	R	R	R
	Norfloxacin(NOR,10 µg)	R	R	R	R	R	R	R	R
Lincosamides	Clindamycin(DA,2 µg)	R	R	R	R	S	R	R	R
Folate pathway inhibitors	Suflamethoxazole-Trimethoprim(SXT 1.25/23.75 µg);	S	R	S	S	R	I	S	S
Phenicols	Chloramphenicol (C,30 µg)	S	S	R	S	S	S	S	R

Ansamycins	Rifampin (RA,5 μg)	R	S	R	R	S	R	R	R
Oxazolidinones	Linezolid(LZD,30 μg)	S	R	R	R	R	R	R	R

*R: Resistant, I: Intermediate, S: sensitive.
